# On the origin of alkali feldspar megacrysts in granitoids. Part 2: evidence for nucleation and growth under magmatic conditions from crystal size distributions of the Cathedral Peak Granodiorite, California, USA

**DOI:** 10.1007/s00410-024-02152-x

**Published:** 2024-06-17

**Authors:** Susanne Seitz, Guilherme A. R. Gualda, Lydia J. Harmon

**Affiliations:** Earth and Environmental Sciences, Vanderbilt University, Nashville, TN 37235 USA

**Keywords:** Crystal size distributions, Alkali feldspar megacrysts, Nucleation and growth, Cathedral Peak Granodiorite, Tuolumne Intrusive Complex

## Abstract

The mechanisms whereby alkali feldspar megacrysts form have been debated for several decades; yet, we do not understand well the processes that lead to their formation. We take advantage of glacially polished outcrop surfaces from the Cathedral Peak Granodiorite in the Tuolumne Intrusive Complex, CA to quantitatively characterize alkali feldspar textures, to provide better insight into their origin. On the glacially polished surfaces, we traced alkali feldspar crystals > 10 mm in the field. From the same localities, we also collected large slabs and stained them to reveal feldspar textures for crystals < 20 mm in size. We scaned the resulting field tracings and rock slabs to quantify CSDs using image processing techniques with the software ImageJ. The CSDs from glacially polished outcrop surfaces and complementary polished and stained rock slabs reveal two stages of crystallization. Crystals > 20 mm show log-linear CSDs with shallow slopes, suggesting magmatic nucleation and growth on timescales of thousands of years. Crystals < 20 mm define a second stage of crystallization, with much steeper slopes, suggesting a period of enhanced nucleation leading to formation of a groundmass during the final stages of solidification on timescales of decades to centuries. We do not find any evidence for CSDs affected by textural coarsening, or any effects of subsolidus processes. Our data suggest that these megacrysts form in large, slowly cooling magma, where low nucleation rates dominate. These crystals are not special in their magmatic formation—only in their size. A change in solidification conditions led to the formation of a groundmass, which warrants further study to better understand this crystallization stage in a plutonic environment.

## Introduction

Large, euhedral alkali feldspar crystals up to 20 cm in size are a common feature in granitoid rocks and have captured the attention of researchers for decades (Vernon 1986; Higgins 1999; Gagnevin et al. 2005; Moore and Sisson 2008; Vernon and Paterson 2008a, b; Johnson and Glazner 2010; Farina et al. 2010; Glazner and Johnson 2013; Barboni and Schoene 2014; Gualda 2019; Chambers et al. 2020). Despite the continued interest, the origin of alkali feldspar megacrysts is still debated, and important questions remain regarding the processes that lead to their formation.

Several textural and compositional characteristics—such as preferred orientation of alkali feldspar megacrysts, their euhedral shapes, the presence of crystallographically-aligned inclusions, and their oscillatory chemical zoning—suggest a magmatic origin (Vernon 1986; Higgins 1999; Moore and Sisson 2008; Vernon and Paterson 2008a, b; Barboni and Schoene 2014; Holness 2018; Gualda 2019; Chambers et al. 2020). However, there is no general agreement about the mechanisms that could lead to the formation of alkali feldspar megacrysts under magmatic conditions. A combination of fast growth and limited nucleation (Swanson 1977; Long 1978) has been invoked to explain the large size of alkali feldspar crystals. Another hypothesis suggests that alkali feldspar megacrysts obtain large sizes by prolonged growth via crystal transfer into different magma batches (Paterson et al. 2016; Holness 2018; Chambers et al. 2020). Textural coarsening (i.e., Ostwald ripening) under magmatic conditions has also been proposed as a mechanism that could play a role in the formation of alkali feldspar megacrysts (Higgins 1999, 2011; Johnson and Glazner 2010).

However, many megacryst-bearing rocks have compositions that suggest sanidine saturates relatively late in the crystallization sequence, after plagioclase and quartz, possibly only after the abundance of solids has reached ≥ 50 wt% (Glazner and Johnson 2013), which has been used as evidence that subsolidus processes play an important role in the formation of alkali feldspar megacrysts, particularly by textural coarsening mechanisms (Glazner and Johnson 2013).

A fundamental—and puzzling—characteristic of rocks bearing alkali feldspar megacrysts is the fact that the megacrysts are much larger than other phases, including plagioclase and quartz. Hence, mechanisms responsible for the origin of alkali feldspar megacrysts need to also explain why plagioclase and quartz do not attain such large sizes as well. Existing theories explain the size difference by lower nucleation rates of alkali feldspar (few large crystals) compared to high nucleation rates of other minerals (more abundant, smaller crystals), though there is no agreement on the specific mechanisms invoked to cause such differences. Higgins (1999) argues that undercooling varies for the different minerals (i.e., suppression of alkali feldspar nucleation). Moore and Sisson (2008) suggest that diffusive barriers during solidification could play a role (i.e., alkali feldspar has the smallest diffusive barrier and thus the slowest nucleation rate compared to other minerals). Kirkpatrick (1983), in contrast, attributes the lower nucleation rate of alkali feldspar to differences in the polymerization of crystals (i.e., alkali feldspar is the most polymerized crystal and thus has slower nucleation rates). Higgins (1999), Johnson and Glazner (2010), and Glazer and Johnson (2013) suggest textural coarsening helps develop large alkali feldspar crystals; however, Gualda (2019) argues that textural coarsening is not an effective mechanism for crystals in the mm to cm scale (see also Holness 2018), and textural coarsening fails to explain why plagioclase and quartz do not attain similar sizes.

In summary, despite the many theories, none appropriately explains the origin of alkali feldspar megacrysts, including the difference in size between the megacrysts and other minerals of the same rock. In this work, we present new textural data on rocks bearing alkali feldspar megacrysts to provide new constraints on their origin.

Crystal size distributions (CSDs) can provide useful information to constrain magmatic processes, being particularly useful in revealing stages and timescales of crystallization (e.g., Cashman 1988, 1992; Cashman and Marsh 1988; Marsh 1988, 1998, 2007; Mock and Jerram 2005; Jerram et al. 2009; Pamukcu et al. 2012, 2020). In this sense, CSDs of alkali feldspar can be used to infer whether observed textures are consistent with magmatic crystallization or not.

The Cathedral Peak Granodiorite—part of the Tuolumne Intrusive Complex (California, USA; Bateman and Chappell 1979)—is characterized by abundant alkali feldspar megacrysts and has been the focus of many detailed studies (e.g., Bateman and Chappell 1979; Higgins 1999; Paterson et al. 2005; Zak and Paterson 2005; Solgadi and Sawyer 2008; Chambers et al. 2020; among many others). We present new data on alkali feldspar CSDs for rocks from the Cathedral Peak Granodiorite, with the aim to gain new insights on alkali feldspar crystallization history and associated timescales, so as to help us understand the origin of alkali feldspar megacrysts in granitoids.

## Geological background

**Fig. 1:**
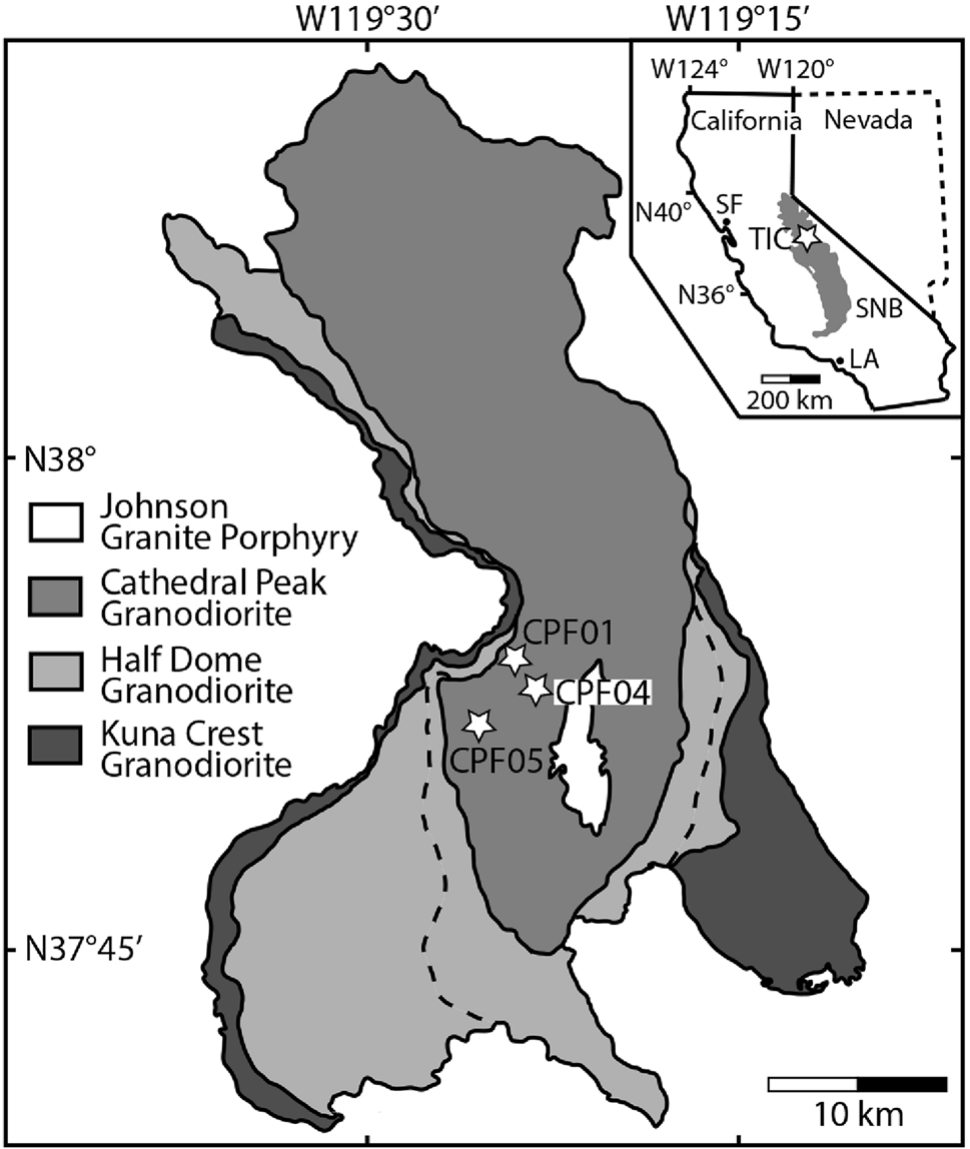
Simplified geological map of the Tuolumne Intrusive Complex, California, USA (after Bateman 1992). The stars mark sample localities of this study. The dashed line divides the outer equigranular Half Dome Granodiorite from the inner porphyritic Half Dome Granodiorite. The inset shows the location of the Tuolumne Intrusive Complex relative to the Sierra Nevada Batholith. *TIC* Tuolumne Intrusive Complex, *SNB* Sierra Nevada Batholith

The Late Cretaceous Tuolumne Intrusive Complex is part of the Sierra Nevada Batholith, California, USA (Fig. 1) (Bateman and Chappell 1979; Bateman 1992). It consists of five concentric intrusive units, with the youngest units preserved towards the center of the complex (Fig. 1). The oldest unit is the Kuna Crest unit (93.5–93.1 Ma; Coleman et al. 2004), a fine to medium-grained, equigranular granodiorite in the east and a tonalite in the west. The next inward unit is the coarse-grained Half Dome Granodiorite. The outer equigranular Half Dome Granodiorite (92.8–91.1 Ma) contains up to 2 cm large hornblende crystals and the inner porphyritic Half Dome Granodiorite (88.8 Ma) is characterized by up to 3 cm long alkali feldspar crystals. The Cathedral Peak Granodiorite (88.1 Ma) appears inward relative to these units, frequently containing up to 20 cm long alkali feldspar crystals (here called megacrysts) in a coarse-grained matrix. The youngest—and innermost—unit in the complex is the Johnson Granite Porphyry (85.4 Ma), a finer grained equigranular granite.

The Cathedral Peak Granodiorite is the most voluminous unit of the Tuolumne Intrusive Complex (Bateman and Chappell 1979). Its composition varies gradually from its edge to its center, with more silicic compositions at the center, towards the contact with the Johnson Granite Porphyry (67.4–74.7 wt%; Bateman and Chappell 1979). Alkali feldspar content varies from more sparsely distributed to locally concentrated clusters and layers (Johnson and Glazner 2010). Alkali feldspar crystals are typically euhedral, they often show Carlsbad twinning (Higgins 1999), and their compositions vary between Or_97_ and Or_76_ (Higgins 1999). Alkali feldspar in the groundmass is normally interstitial to plagioclase and quartz crystals, with an average composition of Or_88_. Alkali feldspar megacrysts are typically exsolved and rich in inclusions of plagioclase, quartz, biotite, titanite, magnetite, hornblende, apatite and zircon (Moore and Sisson 2008). Inclusions are arranged parallel to the oscillatory zones, which primarily reflect variations in Ba concentration (Moore and Sisson 2008). Ba content in alkali feldspar varies between 0.4 and 2 wt% (Higgins 1999; Moore and Sisson 2008). Plagioclase can form crystals up to 1 cm in size. Their composition in the groundmass (An_35_) and as inclusions (An_33_) in alkali feldspar are very similar (Higgins 1999).

## Methods

Over three field sessions, we studied multiple localities within the Cathedral Peak Granodiorite (Fig. 1, Table 1) between Tenaya Lake and Tuolumne Meadows, with the aim of documenting textures and obtaining CSDs of alkali feldspar. The field observations focus on crystals on the cm scale, while we collected large samples to study crystals in the mm to sub-mm scales. For the detailed quantitative textural work, we focused on three areas in which alkali feldspar megacrysts are more sparse and appear more evenly distributed (see Table 1, Fig. 1; for further details, see below).

**Table 1:** GPS coordinates of the studied localities within the Cathedral Peak Granodiorite

Sample	Latitude	Longitude
CPF01	37° 53′ 51.65″ N	119° 24′ 12.44″ W
CPF04	37° 52′ 43.72″ N	119° 23′ 28.47″ W
CPF05	37° 51′ 54.02″ N	119° 25′ 51.26″ W

### Quantitative textural analysis

**Fig. 2:**
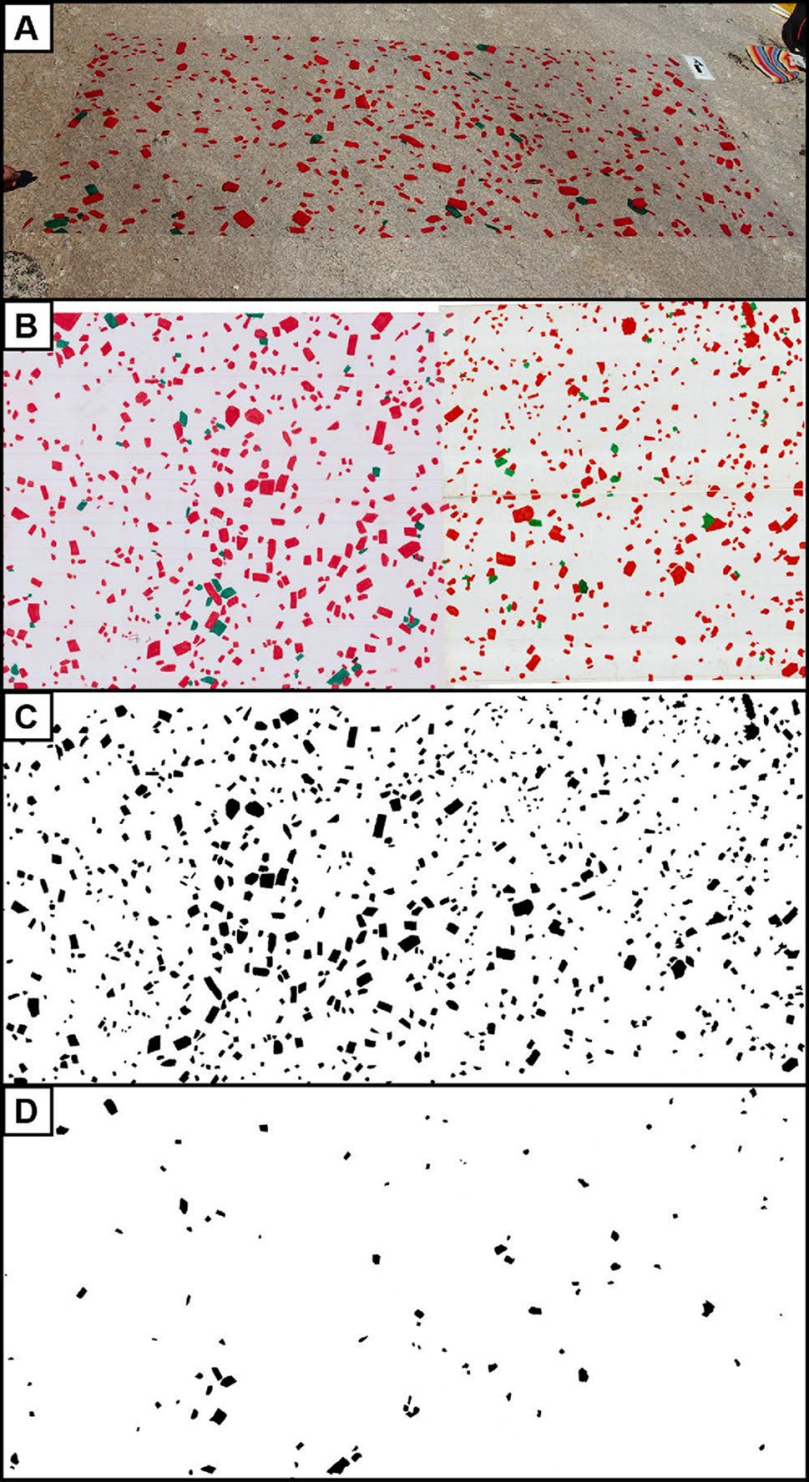
Example of traced alkali feldspar crystals on a glacially polished outcrop surface and resulting scanned images. (A) Alkali feldspar crystals are traced by hand in red and green on an adhesive plastic film attached to the glacially polished surface; arrow in the top right is 10 cm long. (B) Example of a scanned tracing (190 cm long) of alkali feldspar crystals; touching crystals are colored in red and green for image analysis purposes (see text for details). (C) Binary image showing the distribution of red colored alkali feldspar crystals. (D) Binary image showing the distribution of green colored alkali feldspar crystals. Crystal size distribution is calculated using properties of the alkali feldspar crystals obtained from the binary images using the software ImageJ. Data shown are from locality CPF 01

To document alkali feldspar crystals as large as 10–20 cm in size, we traced the outlines of the crystals by hand on transparent contact paper attached to glacially polished outcrop surfaces (Fig. 2A). Our approach is similar to that of Higgins (1999; see also Farina et al. 2010) but allows for measurement of crystal sizes using image processing techniques rather than direct measurement in the field; it also preserves other textural features such as the spatial distribution of crystals. We continued to trace crystals until we could not identify any new crystals with confidence for at least five minutes. The smallest crystals that could be identified with confidence as alkali feldspar and whose outline could be traced on the contact paper are ~ 10 mm in length. It is likely that not all feldspars in size fractions including crystals ≤ 10 mm can be appropriately quantified using the field tracings. We have good confidence, in contrast, that larger crystals were traced and can be appropriately quantified. We chose areas large enough for the field tracings to include approximately 1000 alkali feldspar crystals, so as to ensure the derived CSDs are statistically robust (Mock and Jerram 2005; Gualda 2006). We colored alkali feldspar crystals that are touching on the outcrop surface in different colors (e.g., red, green, and blue; Fig. 2A, B) so that they can be easily separated into different color channels during image analysis (Fig. 2C, D). The field tracings were carefully removed from the outcrop surface and attached to white paper, which was then scanned in color with a resolution of 300 dpi using a large-format scanner.

**Fig. 3:**
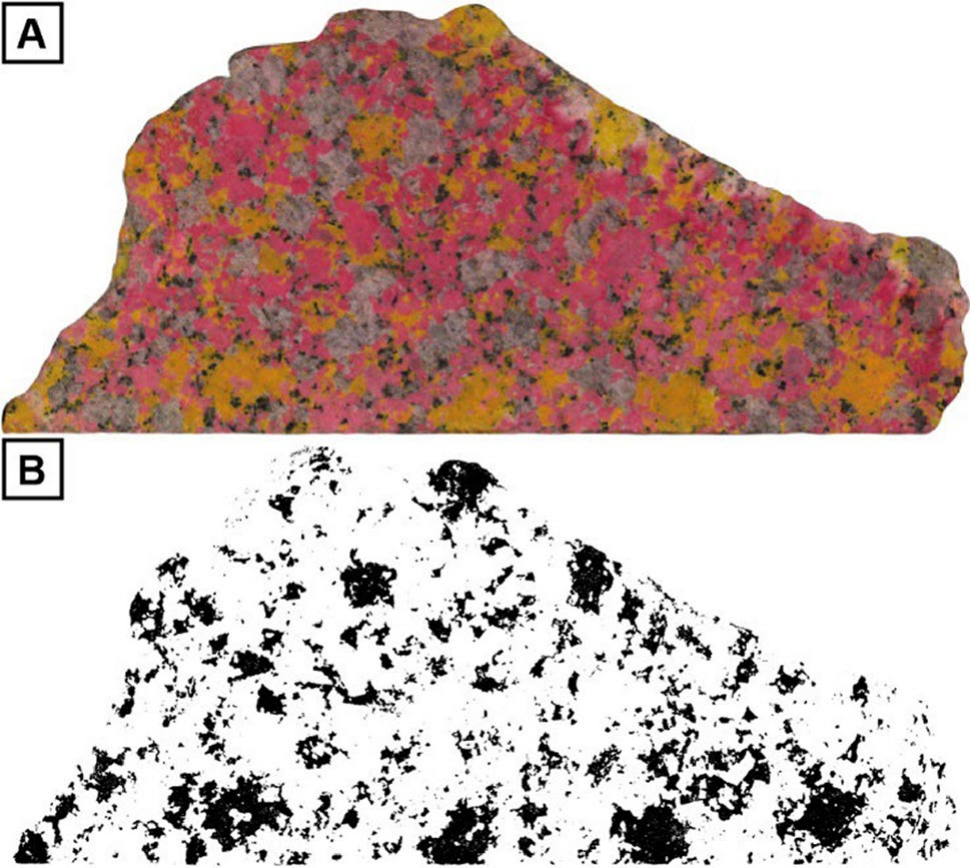
Example of stained rock slab and resulting scanned image. **A** Stained rock slab, with alkali feldspar stained in yellow and plagioclase in pink; quartz is not stained and appears grey; mafic minerals are also not stained and appear black in the image. **B** Binary image showing the distribution of alkali feldspar in black. Crystal size distribution is calculated using properties of the alkali feldspar crystals obtained from the binary image. The sample is 20 cm long

To obtain quantitative textural information for alkali feldspar crystals in the mm to sub-mm scales, we collected large rock samples from the same locations as the field tracings. We collected samples that are dm in size, so that they include enough crystals in size fractions ≤ 10 mm that cannot be quantified using the field tracings. Samples were cut with a diamond blade, polished to remove saw marks, and then stained to distinguish between alkali feldspar and plagioclase (Fig. 3A). Prior to staining, each rock slab was etched with 50% HF for 30 s and rinsed; the staining was conducted in two steps. To stain alkali feldspar, the rock slabs were immersed for 60 s in a 50% sodium cobaltinitrite solution. To stain plagioclase, the slabs were immersed for 30 s in a 20% amaranth solution. The samples were rinsed with water and immersed in a barium chloride solution to set the staining between staining steps. The stained rock slabs were then scanned in color with a resolution of 600 dpi using a flatbed scanner.

The scanned tracings (Fig. 2B) and rock slabs (Fig. 3A) were processed and analyzed with the image analysis software ImageJ (Schneider et al. 2012). Scanned tracings (Fig, 2B) were split into red, green, and blue color channels to separate touching alkali feldspar crystals. A threshold was then applied to select alkali feldspar megacrysts in the red and green color channels and scans were then converted into binary images (Fig. 2C, D). Groundmass alkali feldspar was separated from plagioclase in the stained and scanned rock slabs (Fig. 3A) by choosing a color threshold corresponding to stained alkali feldspars, which resulted in binary images of only alkali feldspars (Fig. 3B). Touching alkali feldspar crystals cannot be separated by this technique, and therefore there could be some bias towards larger crystal sizes in the slab data. The smallest crystals measured in the rock slabs are 0.3 mm in size, which corresponds to 7 pixels in the scanned images. The binary images of the tracings and rock slabs were used to obtain—also using ImageJ—the properties of alkali feldspar crystals, including length, width, the area occupied by the crystals, the orientation, and the centroid of the crystals.

### Stereological corrections

We use the program CSDCorrections (Higgins 2000, 2006) to perform stereological corrections and to calculate resulting CSDs. CSDCorrections includes corrections for (1) the intersection probability effect—smaller crystals are less likely to be intersected by the studied section; and (2) the cut section effect—crystals are unlikely to be cut along their central section, typically yielding sections that are smaller than their true size (Sahagian and Proussevitch 1998; Higgins 2000). The intersection probability effect can be corrected by dividing the number of crystals per unit area by the mean size of each bin size interval. We explore the sensitivity of the main parameters needed to calculate CSDs by varying the size interval (or bin size).

The nature of the fabric and the crystal shape must be known to correct for the cut section effect (Higgins 2000; Morgan and Jerram 2006). In the areas we studied in detail, alkali feldspar crystals appear mainly sparsely distributed, so we assume a massive (i.e., non-foliated) fabric for the stereological corrections; even if a weak fabric is observed, its effect on the resulting CSDs would be negligible. To directly measure the three-dimensional shape of the alkali feldspar crystals, we collected whole alkali feldspar megacrysts from lightly weathered Cathedral Peak Granodiorite, from which whole alkali feldspar megacrysts can easily be dislodged from surrounding matrix. Long, intermediate, and short dimensions of 40 whole alkali feldspar crystals were measured with a caliper. Additionally, we employ the program CSDslice (Morgan and Jerram 2006) to estimate the grain shapes of both megacrystic and groundmass alkali feldspar. Estimates obtained with CSDslice for the megacrysts are compared with the results from direct measurement of whole megacrysts collected in the field.

### Combination of data from field tracings and rock slabs

Data are plotted on semi-logarithmic diagrams of population density versus crystal size, where population density corresponds to the number of crystals per bin size per volume (see Marsh 1988, 1998).

**Fig. 4:**
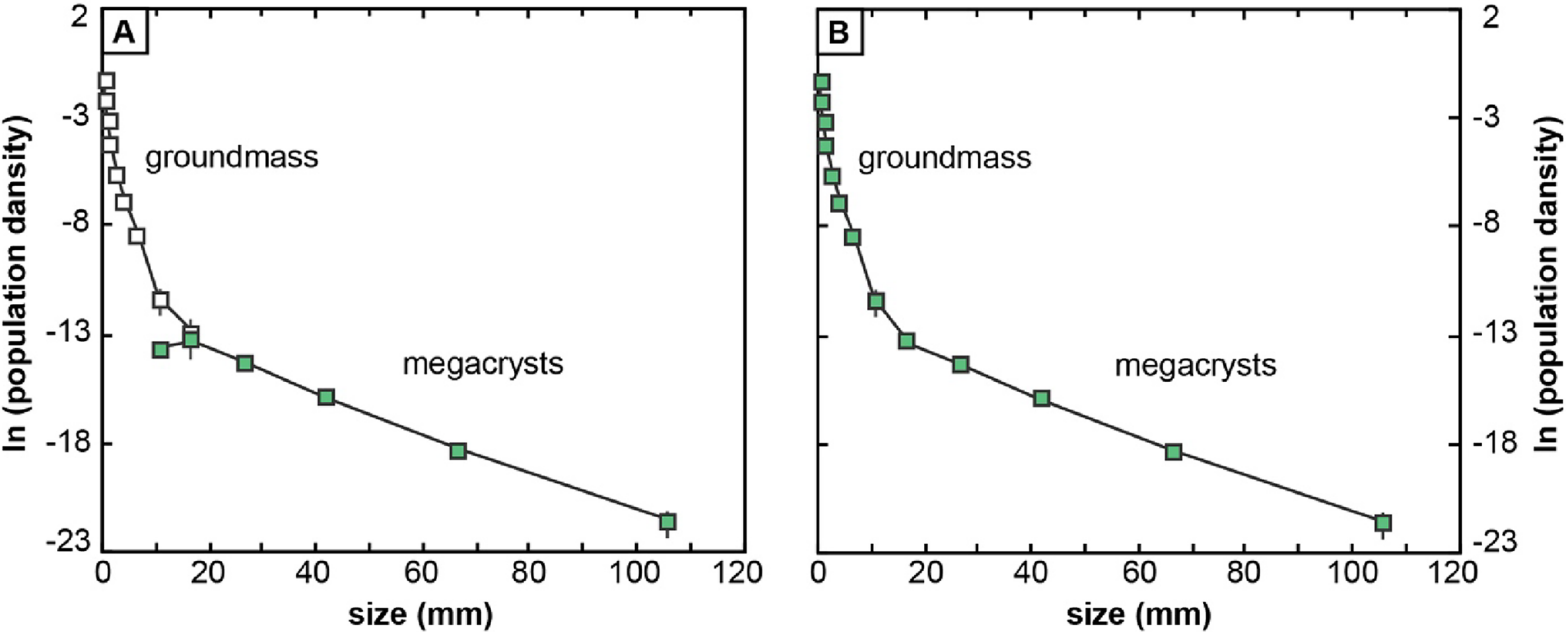
Illustration of procedure used to obtain CSDs over a large range of crystal sizes using information from field tracings and rock slabs. **A** CSD results derived from field tracing are shown as filled green squares; CSD results derived from rock slab are shown as open squares. **B** Combined results, taking advantage of the excellent overlap between the two approaches for the grain size between 10 and 20 mm (see text for details), resulting in a continuous CSD over a large range of crystal sizes. Data for crystals > 20 mm come from the tracings and for crystals < 20 mm data come from the stained rock slabs. Results shown are for sample CPF 01

To generate reliable CSDs spanning crystal sizes from sub-mm up to 20 cm, we combine results obtained using the field tracings and stained rock slabs into a single CSD (see Higgins 1999; see also Gualda and Rivers 2006; Jerram et al. 2009; Pamukcu and Gualda 2010). Data from the field tracings are used for large crystals (i.e., > 10 mm), while data from the stained slabs are used for smaller crystals (Fig. 4). There is good agreement between the results from both methods for the largest bin size in the slab (Fig. 4A), showing that (1) field tracings cannot be used to quantify crystals ≤ 10 mm, as expected; (2) the slabs are large enough to include enough crystals ~ 10 mm to properly quantify that size fraction.

### Calculation of crystallization times

We derive crystallization times for alkali feldspar megacrysts using the formalism of Marsh (1988), which states that, in a log-linear diagram of population density versus crystal size, the slope corresponds to − 1/(G * τ) where G is the growth rate and τ is the residence time (see Marsh 1988). The growth rate (G) needs to be known to calculate the crystallization times. While alkali feldspar growth rates derived from experiments vary widely from 10^–8^ to 10^–15^ m/s (Swanson 1977; Long 1978), results for pre-eruptive crystallization of quartz and feldspar in natural silicic systems (Cashman 1988; Davidson et al. 2001; Gualda et al. 2012, 2018; Barboni and Schoene 2014; Pamukcu et al. 2015; Pitcher et al. 2021) suggest a smaller range of growth rates between 10^–12^ and 10^–14^ m/s for megacrystic alkali feldspar, and growth rates between 10^–10^ and 10^–14^ m/s for groundmass alkali feldspar (see discussion for details).

## Results

### Field observations

**Fig. 5:**
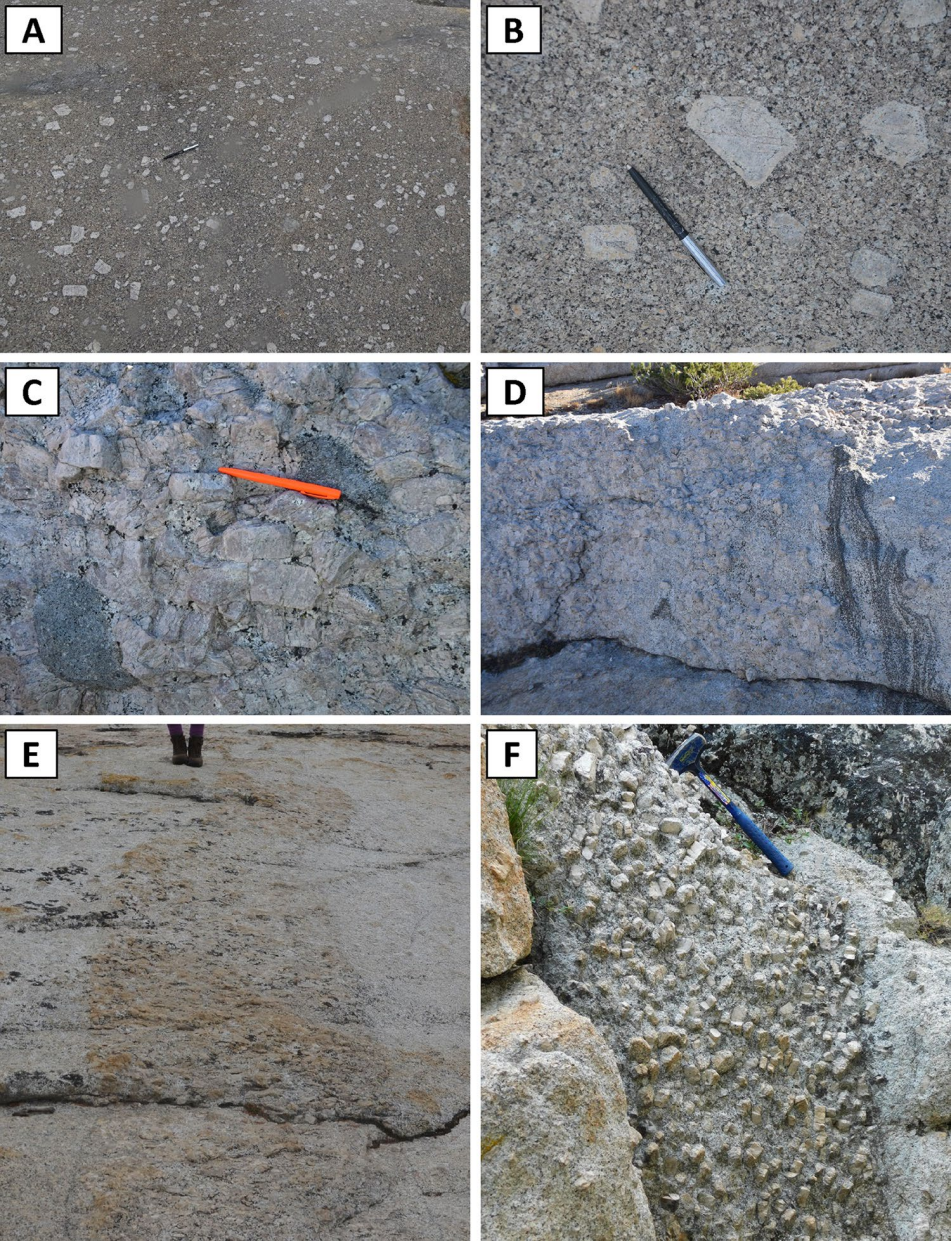
Images showing typical magmatic textures of the Cathedral Peak Granodiorite as observed in the field. **A** Large alkali feldspar crystals are manly distributed in a sparse manner, with no preferred orientation. **B** Detailed view showing the continuous range of sizes of alkali feldspar megacrysts; note that the number of crystals of a given size decreases with increasing size. **C, D** Concentrations of alkali feldspar megacrysts; note that crystals are primarily of similar size, and groundmass is very low in abundance or entirely lacking. **E, F** Tabular bodies with high concentration of alkali feldspar megacrysts; note the sharp contacts with host rock, which shows dispersed alkali feldspar megacrysts. The pens in all images are 13 cm long

We focused much of our field work on glaciated surfaces that expose large areas of uninterrupted outcrops. While there is some variability along those surfaces, we find that there are large, homogeneous areas in which alkali feldspar megacrysts are not particularly abundant, and they appear dispersed in a finer-grained matrix. In most of these areas, we see no evidence of preferred orientation of the alkali feldspar megacrysts (Fig. 5A, B). Further, examination of these areas shows that the number of megacrysts of a given size decreases with increasing size, with no gap in crystal abundance between megacrysts and groundmass crystals (Fig. 5A). In other words, the alkali feldspar grain size variations in these areas are more characteristic of seriated—rather than porphyritic—textures.

**Fig. 6:**
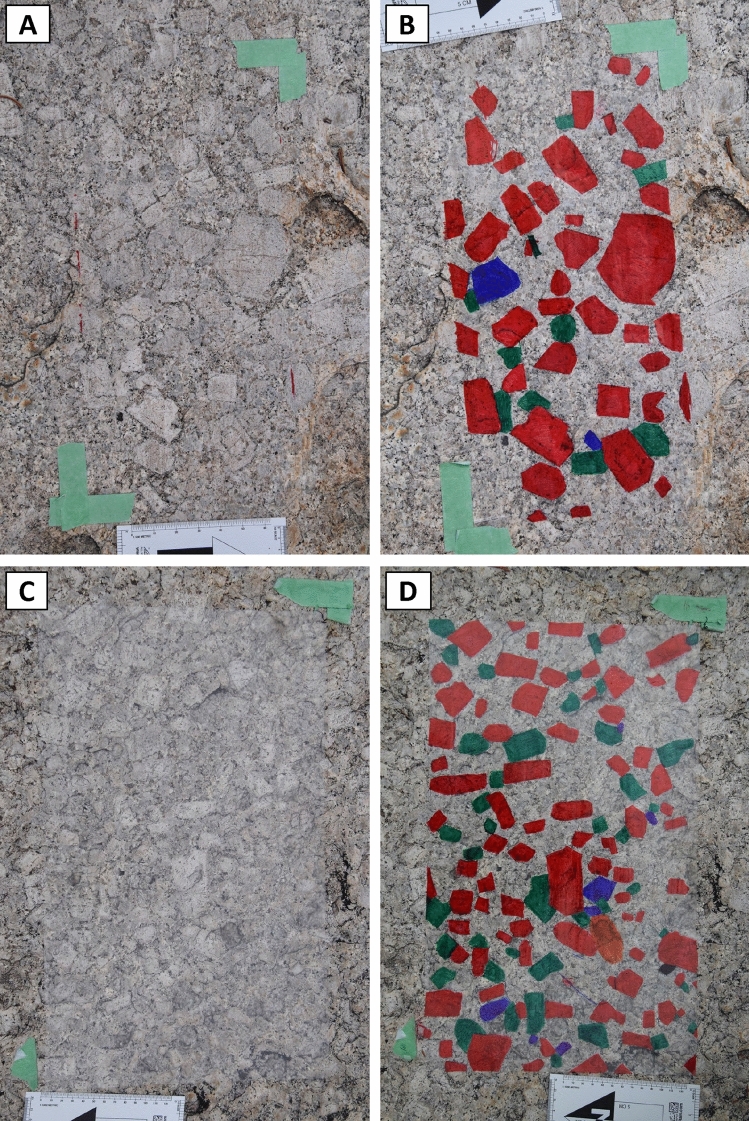
Examples of regions with concentrations of alkali feldspar megacrysts without sharp boundaries with adjacent rock. **A, C** Field photographs showing the plastic film adhered to the rock, prior to tracing. **B, D** Field photographs after tracing. Note that, in both examples, megacrysts are more abundant than in areas with dispersed megacrysts. Importantly, the number of crystals of a given size decreases with increasing crystal size. Scale in centimeters visible in all photos

We also see areas in which alkali feldspar megacrysts are highly concentrated (Figs. 5C–F, 6). In some of these areas, there is a sharp contact between the highly concentrated megacryst-bearing rock and the adjacent rock with more dispersed megacrysts. In the field, some of these concentrated areas appear as tabular bodies of variable orientation (Fig. 5C–F), with widths of cm to dm and lengths that extend from m to dam (Fig. 5E, F). In other locations, the local clusters with higher concentration of alkali feldspar megacrysts lack clear boundaries with the surrounding rock that has more dispersed megacrysts (Fig. 6); our observations suggest that these portions range in area from a few m^2^ to tens of m^2^, but more work is necessary to properly characterize them in greater detail, especially as the third dimension of these bodies is, as yet, unclear.

### Crystal size distributions

**Fig. 7:**
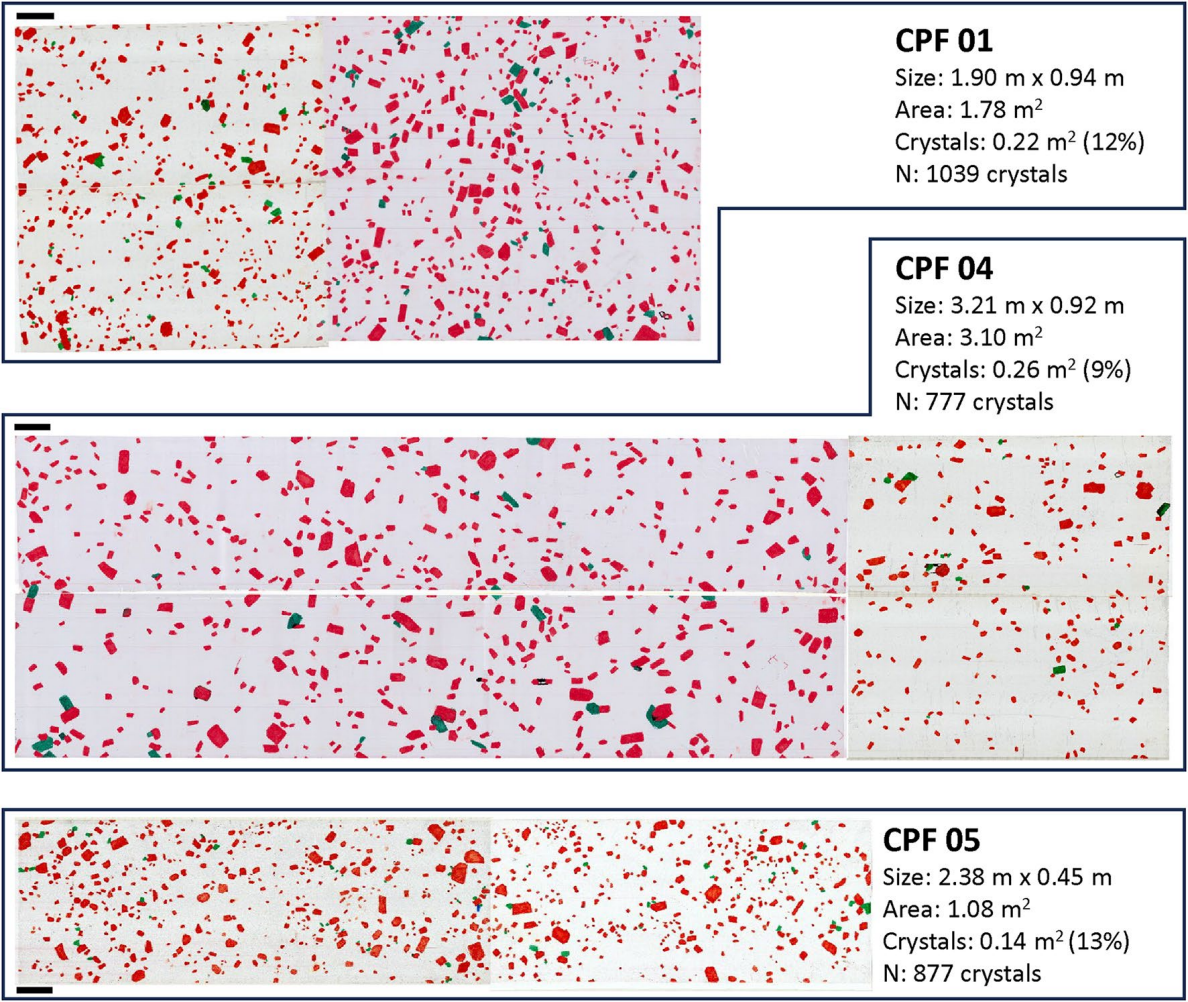
Scanned images of field tracings for the three outcrops studied here. Size and area of the analyzed regions are given, as well as the area occupied by crystals and the number of crystals. Black bars next to each tracing correspond to 10 cm

**Fig. 8:**
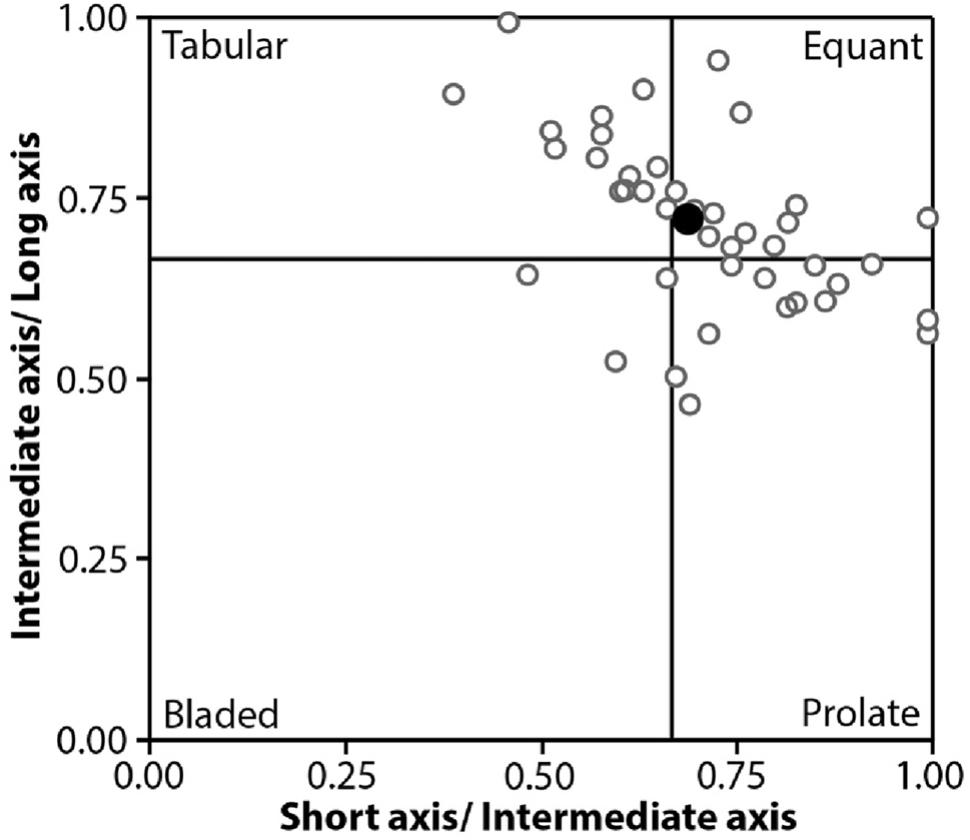
Diagram showing the distribution of alkali feldspar shapes from the Cathedral Peak granodiorite. The black dot corresponds to the axial average ratio of 1:1.5:2

**Fig. 9:**
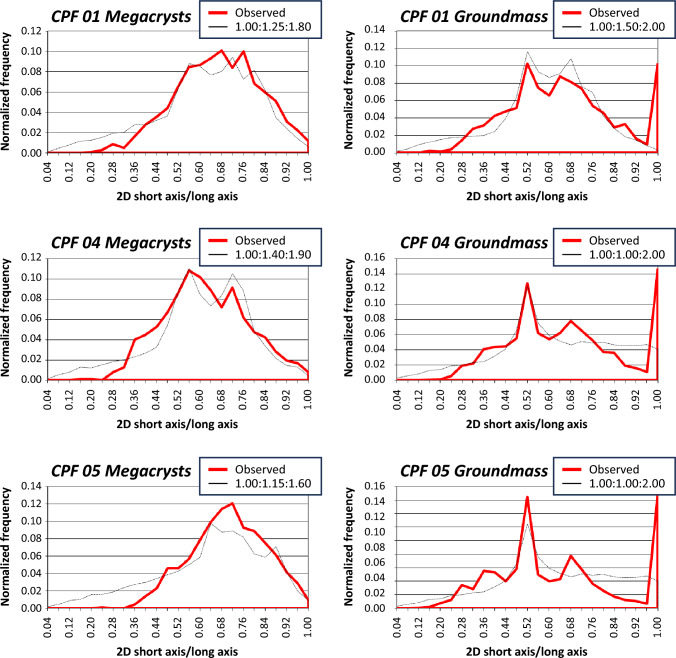
Results of application of the program CSDslice to obtain best-fit axial ratios for alkali feldspar megacrysts and groundmass. Note the similarity between all the results

The large areas we traced span from ~ 1 to ~ 3 m^2^ in area, with the total number of crystals ranging from 777 to 1039; alkali feldspar crystals correspond to 9–13% of the traced area (Fig. 7). Direct measurements of 40 whole, individual alkali feldspar megacrysts collected in the field yield an average axial ratio of 1:1.5:2 (Fig. 8). Results from CSDslice suggest megacrystic axial ratios of 1:1.25:1.8 for CPF 01, 1:1.4:1.9 for CPF 04, and 1:1.15:1.6 for CPF 05, while groundmass results suggest axial ratios of 1:1.5:2 for CPF 01. 1:1:2 for CPF 04, and 1:1:2 for CPF 05 (Fig. 9). The axial ratios obtained by direct measurement of whole megacrysts are thus very similar to those estimated using CSDslice, and they show only small differences between megacrysts and groundmass (likely within error of the estimates using CSDslice). Thus, for simplicity, we use the axial ratios obtained from direct measurement of megacrysts (i.e., 1:1.5:2) for stereological corrections of all of our data using CSDCorrections (see Methods).

**Fig. 10:**
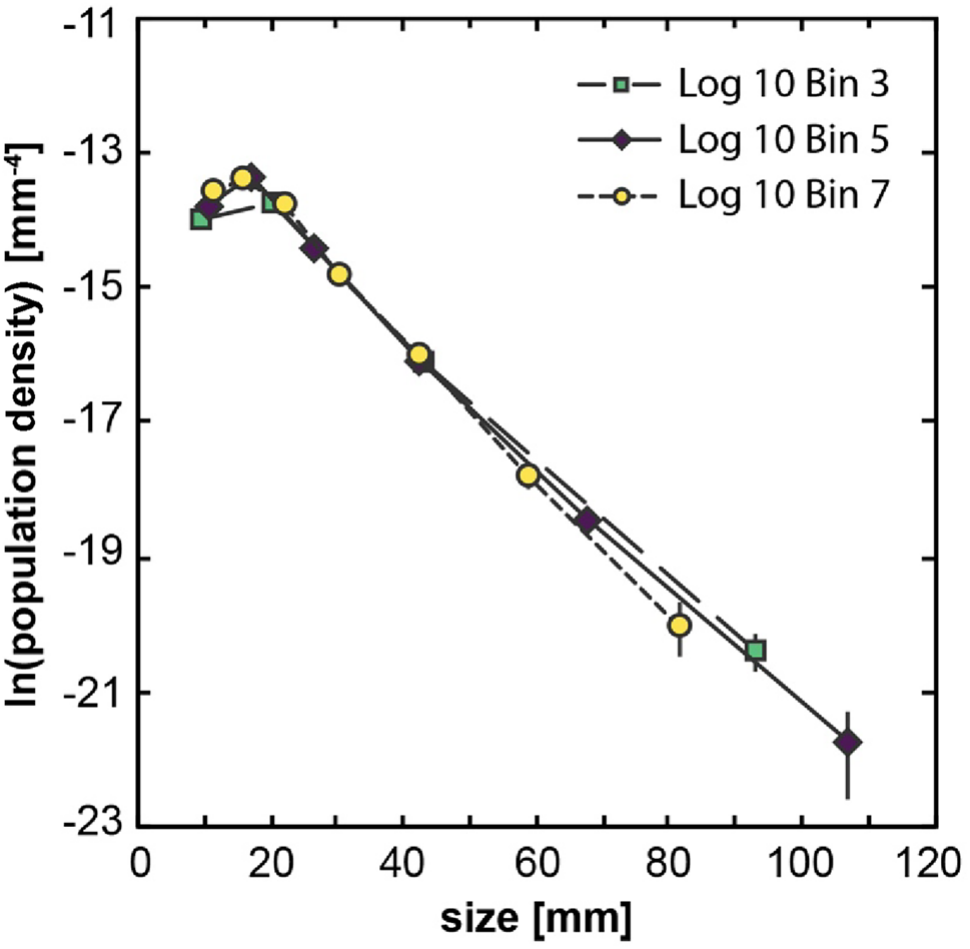
Example of the effect of bin size intervals used by CSDCorrections on the resulting CSD of sample CPF 01. The CSDs for the different bin sizes are almost identical

Use of CSDCorrections also requires making a choice of bin size used for the stereological correction calculations (see Methods). To explore the effect of bin size on the slope of the resulting CSDs (Fig. 10), we calculated CSDs with logarithmic size intervals of 10^0.33^ (bin 3 option in CSDCorrections), 10^0.20^ (bin 5) and 10^0.14^ (bin 7) at a fixed axial ratio of 1:1.5:2. For all bin sizes used, we see a reduction in the population density values for bin sizes < 15 mm—we attribute this reduction to our inability to trace all alkali feldspar crystals of such sizes in the field, and thus interpret this to be an artifact of our method (see Methods). The resulting slopes (for bin sizes > 15 mm) vary between − 0.089 and − 0.098 mm^−1^, showing that the choice of bin size is minor and can be effectively neglected for the purposes of this study. For simplicity, we choose an intermediate bin size interval (bin 5) to calculate CSDs for the 3 samples studied here.

**Fig. 11:**
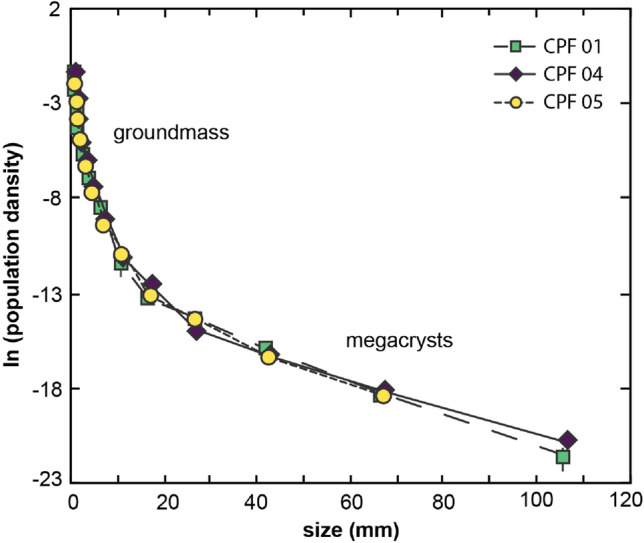
Combined CSDs of megacryst and groundmass alkali feldspar. Note the similarities between the CSDs of different samples

The resulting CSDs—showing the combination of megacryst data from the field tracings and groundmass data from rock slabs—are all very similar to each other (Fig. 11). The CSDs can be described as kinked, and they can be divided into two domains: large crystals and megacrystic alkali feldspar (hereafter simply “megacrysts”), with the long dimension larger than 20 mm; groundmass alkali feldspar, with crystal sizes below 20 mm (Fig. 11).

### Crystallization times

Because the resulting CSDs are kinked (Fig. 11), we calculate different slopes for the groundmass and megacrystic portions (Table 2). Using growth rates between 10^–12^ m/s and 10^–14^ m/s (see discussion for further details), we obtain crystallization times for megacrystic alkali feldspar that vary from a few hundred years (i.e., 0.29–0.45 ka) to a few tens of thousands of years (i.e., 29–45 ka; see Table 3). For groundmass alkali feldspar, using growth rates of 10^–10^ to 10^–14^ m/s (see discussion for further details), we obtain crystallization times of months (i.e., 0.23–0.32 a) to a few thousands of years (i.e., 2.3–3.2 ka; see Table 4).

**Table 2:** Slopes of crystal size distributions for megacrystic and groundmass alkali feldspar for the different samples

	CSD slope (mm^−1^)		
	CPF01	CPF04	CPF05
Megacrysts	− 0.097	− 0.070	− 0.111
Groundmass	− 1.37	− 0.985	− 1.23

**Table 3:** Growth time of alkali feldspar megacrysts (crystal size > 20 mm)

Growth rate (m/s)	Growth time (ka)*		
	CPF01	CPF04	CPF05
10^–12^	0.33	0.45	0.29
10^–13^	3.3	4.5	2.9
10^–14^	33	45	29

*Growth time (τ) is given by τ = – 1/(G*m), where m is the slope of the CSD (Marsh 1988)

**Table 4:** Growth time of alkali feldspar groundmass (crystal size < 20 mm)

Growth rate (m/s)	Growth time (a)*		
	CPF01	CPF04	CPF05
10^–10^	0.23	0.32	0.26
10^–12^	23	32	26
10^–14^	2.3*10^3^	3.2*10^3^	2.6*10^3^

*Growth time (τ) is given by τ = – 1/(G*m), where m is the slope of the CSD (Marsh 1988)

## Discussion

Alkali feldspar megacrysts are a common feature of granitoid rocks, but the roles played by magmatic versus subsolidus processes have been debated over several decades (Vernon 1986; Higgins 1999; Moore and Sisson 2008; Vernon and Paterson 2008a, b; Johnson and Glazner 2010; Glazner and Johnson 2013; Barboni and Schoene 2014; Gualda 2019; Chambers et al. 2020). Several lines of evidence, including the nature of the preferred orientation in some outcrops, the ubiquitous euhedral shape, the alignment of mineral inclusions according to crystallographic directions, the oscillatory zoning, and the growth at typical magmatic temperature deduced from mineral inclusions, have been used as evidence for a magmatic origin of alkali feldspar megacrysts (Bateman and Chappell 1979; Vernon 1986; Higgins 1999; Paterson et al. 2005; Zak and Paterson 2005; Moore and Sisson 2008; Vernon and Paterson 2008a; Holness 2018). The alkali feldspar CSDs presented here contain important information about the crystallization history of these magmas. For minerals formed from a melt, the theory of CSDs predicts an exponential distribution of population density versus crystal size, resulting in log-linear CSDs (Marsh 1988, 1998). A sudden change in nucleation rates is usually invoked to explain kinked CSDs as any change in the nucleation rate causes a change in slope of the CSD (Cashman 1988, 1992; Marsh 1998). Below, we discuss the crystallization stages, associated timescales of crystallization, and alkali feldspar sizes.

### Crystallization stages

The CSDs presented here can be divided into a megacryst part and a groundmass part, with a clear change in slope between them (Fig. 11) (see also Higgins 1999; Farina et al. 2010). The megacryst CSDs are characterized by log-linear shapes, shallow slopes, and large crystal sizes (Fig. 11). These megacryst CSDs can be interpreted as the result of a single nucleation event (e.g., Marsh 1988, 1998), which is compatible with largely uninterrupted, continuous crystallization of alkali feldspar in a large magma body such as the Cathedral Peak Granodiorite. The shallow slope of the megacryst CSDs and the large crystal sizes of the megacrysts indicate prolonged growth, in agreement with the slow cooling of a pluton (e.g., Gualda et al. 2004; Pamukcu et al. 2012).

Literature data on Zr-in-titanite temperatures from alkali feldspar-hosted titanite inclusions show that temperature during growth of alkali feldspar megacrysts was buffered at around 740 °C (Moore and Sisson 2008). Previous studies argue that buffered temperatures suppress alkali feldspar nucleation and promote textural coarsening, which leads to megacryst formation (Higgins 1999; Johnson and Glazner 2010; Glazner and Johnson 2013). Textural coarsening leads to hump-shaped CSDs, characteristically log-normal in shape (Higgins 1999; Kile et al. 2000). Importantly, the megacryst CSDs presented here lack such hump-shaped distributions (Fig. 11); consequently, we rule out the possibility that coarsening played a role in the formation of alkali feldspar megacrysts. This is in agreement with the conclusions of Gualda (2019), who demonstrates that the timescales required to generate alkali feldspars by coarsening are prohibitively large in the case of mm to cm-sized crystals (see also Holness 2018).

Finally, trace-element compositions from Chambers et al. (2020) show that megacryst-hosted zircon crystals and groundmass zircon crystals did not crystalize from melts with the same composition; the low Zr/Hf ratio of groundmass zircon crystals suggests that the melt had previously crystalized a significant amount of zircon (see Claiborne et al. 2006), such that alkali feldspar megacrysts are not the result of coarsening at low melt percentages. Megacryst CSDs presented here, in combination with existing data on megacryst-hosted inclusions from the literature thus favor growth of megacrysts under magmatic conditions, without significant influence of textural coarsening processes.

The groundmass CSDs presented here are also characterized by log-linear shapes, but with much steeper slopes, with very high population densities for small crystal sizes (Fig. 11). The change in slope between megacryst and groundmass CSDs indicates that alkali feldspar nucleation rates were substantially higher during groundmass formation (see, for instance, Marsh 1988, 1998). The small size of groundmass alkali feldspar crystals is a direct result of higher nucleation rates, as the resulting higher number density of alkali feldspar crystals limits the maximum size of individual crystals formed during this stage (Marsh 1998). Given the positive correlation between nucleation rate and undercooling (e.g., Cashman 1988, 1993), we conclude that groundmass alkali feldspar must have formed during a period of increased undercooling. In agreement, Zr-in-titanite temperatures from groundmass titanite grains give lower temperatures, indicating lower temperatures during growth of alkali feldspar groundmass when compared to the megacrysts (Moore and Sisson 2008). However, other factors, such as volatile contents, could lead to increased undercooling, and we do not currently understand the change in conditions that led to increased nucleation rates during groundmass crystallization. This is an area that deserves more detailed studies.

### Timescales of alkali feldspar growth

The CSDs of alkali feldspar megacrysts are compatible with crystallization in a large magma body over a prolonged time, while the steeper CSDs for groundmass crystals reveal increased nucleation rates towards the end of solidification of Cathedral Peak Granodiorite magmas. To constrain crystallization times for alkali feldspar megacrysts and groundmass from CSDs, it is necessary to constrain relevant growth rates for alkali feldspar megacrysts and groundmass.

Experiments demonstrate that very fast growth rates (10^–8^–10^–12^ m/s) for alkali feldspar in granitic magmas are possible (Swanson 1977; Long 1978). It is, however, unlikely that such high growth rates can be attained during cooling of large bodies of granitic magma, given that cooling rates are limited by the high volume over area ratios of these magma bodies. In volcanic systems, fast growth rates—on the order of 10^–7^–10^–9^ m/s—are often associated with decompression-driven, syn-eruptive growth of minerals such as feldspar and quartz (e.g., Cashman 1988; Gualda and Sutton 2016; Pamukcu et al. 2016; Befus and Andrews 2018; and references therein). It is highly unlikely that such fast growth rates could be sustained at depth in a magmatic system of the dimensions of the Cathedral Peak Granodiorite during cooling.

Studies of silicic volcanic rocks further constrain growth rates for feldspar and quartz phenocrysts. Growth rates of alkali feldspar, plagioclase and quartz are notably similar, at least on an order of magnitude scale. Alkali feldspar growth rates have been estimated to be between 10^–13^ and 10^–14^ m/s (Davidson et al. 2001); plagioclase growth rates have been found to be between 10^–13^ and 10^–14^ m/s (Cashman 1988); and quartz growth rates have been found to be between 10^–8^ and 10^–14^ m/s, with typical growth rates for pre-eruptive growth on the order of 10^–12^ to 10^–13^ m/s (Pamukcu et al. 2015, 2016; Seitz et al. 2016; Gualda and Sutton 2016; Pitcher et al. 2021). Gualda et al. (2012) argue that, in most volcanic rocks, alkali feldspar, plagioclase, and quartz have generally similar sizes, which suggests that their growth rates are similar during pre-eruptive conditions, consistent with the independently estimated growth rates discussed above. Irrespective of whether a specific granitic magma body did or did not feed eruptions, the conditions of pre-eruptive growth recorded by volcanic rocks are likely generally a good proxy for the conditions of crystallization of most granite magmas stored at shallow crustal levels. Consequently, growth rates between 10^–12^ and 10^–14^ m/s seem reasonable for the growth of alkali feldspar megacrysts of the Cathedral Peak Granodiorite.

Finally, previous studies using zircon geochronology can also be used to constrain alkali feldspar growth rates in the Cathedral Peak Granodiorite. Barboni and Schoene (2014) suggest growth times of alkali feldspar megacrysts on the order of a few tens of thousands of years (Barboni and Schoene 2014), while Chambers et al. (2020) suggest growth times on the order of ~ 500 ka (Chambers et al. 2020). Taking into account the sizes of the crystals analyzed by Barboni and Schoene (2014) and by Chambers et al. (2020), we use these growth times to estimate growth rates of 10^–14^ to 10^–15^ m/s. Growth rates of 10^–15^ m/s are likely too slow for alkali feldspar, given that they approach growth rates inferred for zircon crystallization (e.g., Watson 1996); in other words, alkali feldspar growth rates cannot be as slow as zircon growth rates, given the contrast in crystal size between alkali feldspar (up to 10 s of cm in size) and zircon (typically < 1 mm in size). Also, the samples used by Chambers et al. (2020) are from the contact between the Cathedral Peak Granodiorite and the Half Dome Granodiorite. The contact between these two units is gradational over several tens to hundreds of meters, suggesting prolonged interaction between different magma types (Chambers et al. 2020), which could have led to growth of alkali feldspar megacrysts over more extended periods of time than typical of Cathedral Peak Granodiorite located away from the contact. We thus conclude that minimum growth rates for alkali feldspar megacrysts are on the order of 10^–14^ m/s, consistent with our estimates based on pre-eruptive growth of major felsic minerals (10^–12^–10^–14^ m/s; see above).

We argue above that growth of alkali feldspar groundmass in the Cathedral Peak Granodiorite took place under conditions of increased undercooling, as revealed by the high number density of alkali feldspar in the groundmass. As such, we expect that growth rates would be potentially higher during alkali feldspar groundmass growth. It is unlikely that growth rates as fast as those observed during eruptive decompression recorded in volcanic rocks could be attained in plutonic systems. We thus consider growth rates on the order of 10^–10^–10^–14^ m/s for growth of the alkali feldspar groundmass.

Growth times of alkali feldspar megacrysts presented here (Table 3) vary between 0.29 and 0.45 ka for a growth rate of 10^–12^ m/s to 29–45 ka for a growth rate of 10^–14^ m/s, which places the crystallization times of tens of thousands of years suggested by growth rates of 10^–14^ m/s as upper bounds to the timescales of alkali feldspar megacryst formation. In this sense, our CSDs of alkali feldspar megacrysts are consistent with growth under magmatic conditions in timescales of thousands of years, without the need for special processes to form megacrysts up to 20 cm in size. Furthermore, our timescales are consistent with evidence from zircon inclusions in alkali feldspar crystals (Barboni and Schoene 2014; Chambers et al. 2020), as well as with the current understanding of pre-eruptive crystal growth in volcanic rocks (Cashman 1988; Davidson et al. 2001; Pamukcu et al. 2015, 2016; Seitz et al. 2016; Pitcher et al. 2021).

For groundmass alkali feldspar, the slopes of the CSDs are significantly steeper (see Table 2), suggesting shorter growth times. For the same growth rates used for the megacrysts, we get growth times shorter than a few thousand years (Table 4). However, the change in slope suggests a crystallization stage in which nucleation rate was higher than during megacryst growth (see above). This would be accompanied by faster growth rates, suggesting that groundmass alkali feldspar crystallization timescales are likely shorter, probably shorter than a few hundred years, and possibly only a few decades long (see Table 4).

### Constraints on the origin of alkali feldspar megacrysts

The contrast in size between alkali feldspar and other minerals in megacrystic Cathedral Peak Granodiorite is striking. It is difficult to explain why only alkali feldspar forms crystals up to 20 cm in size. Three different hypotheses have been invoked to explain the formation of alkali feldspar megacrysts: (1) textural coarsening, (2) prolonged growth, or (3) fast growth rates in combination with slow nucleation rates.

Timescales of coarsening (Gualda 2019), zircon compositions (Chambers et al. 2020), and the CSDs presented here all suggest that coarsening is not the mechanism responsible for the formation of alkali feldspar megacrysts. For the Cathedral Peak Granodiorite, CSDs for alkali feldspar megacrysts are consistent with largely continuous growth under low nucleation in a slow cooling pluton.

Importantly, it has generally been inferred that the large alkali feldspar crystal sizes imply fast growth rate. However, the key textural characteristic of the Cathedral Peak Granodiorite is the small number density of alkali feldspar megacrysts. This suggests that the main textural control during solidification of Cathedral Peak magmas was, in fact, low nucleation rates (see Marsh 1998; Zieg and Marsh 2002) rather than fast growth rates. This suggests low degrees of undercooling, at least prior to groundmass formation. In this sense, fast growth rates are not necessary to form alkali feldspar megacrysts. Our data suggest growth of alkali feldspar megacrysts under small degrees of undercooling, corresponding to conditions in which cooling is slow enough to inhibit alkali feldspar nucleation. The mechanisms that led to lower crystal number densities (and, thus, inferred nucleation rates) for alkali feldspar megacrysts, when compared to plagioclase and quartz, are not yet well understood, and deserve further study.

Our CSDs reveal that a nucleation event took place during groundmass formation, suggesting a shift in crystallization conditions towards conditions that led to higher nucleation rates. Given the thermal inertia of large bodies of magma, it is unlikely that this represents a change in cooling rate, suggesting a potential role for decompression or changes in volatile content. However, further work is necessary to properly constrain the conditions that led to the change in crystallization conditions leading to groundmass formation.

## Conclusions

The origin of alkali feldspar megacrysts in granitoids has been a matter of debate for more than a century. Recent work has emphasized the potential importance of late-magmatic and subsolidus processes on their origin (Higgins 1999; Johnson and Glazner 2010; Glazner and Johnson 2013). We present here detailed textural characterization of alkali feldspar textures from the Cathedral Peak Granodiorite, the most voluminous unit of the Tuolumne Intrusive Complex.

Field evidence suggests that much of the Cathedral Peak Granodiorite is characterized by rocks with dispersed alkali feldspar megacrysts. Local concentrations of alkali feldspar megacrysts are common, sometimes forming tabular features with sharp contacts with surrounding rock, but they represent a small volume when compared to granodiorite with dispersed feldspars.

The CSDs for alkali feldspars we derive from glacially polished outcrop surfaces and complementary polished and stained rock slabs in regions where alkali feldspars are sparsely distributed reveal two stages of crystallization. Crystals > 20 mm show log-linear CSDs with shallow slopes, which suggest continuous growth by magmatic nucleation and growth under conditions of slow cooling at low degrees of undercooling. We do not find any evidence for CSDs affected by textural coarsening, consistent with the predictions made by Holness (2018) and Gualda (2019). Crystals < 20 mm define a second stage of crystallization, with much steeper CSD slopes, suggesting a period of enhanced nucleation leading to formation of a groundmass during the final stages of solidification of the Cathedral Peak Granodiorite.

Using growth rates estimated for alkali feldspar and other felsic phases from the literature, we estimate that alkali feldspar megacrysts grew on timescales of thousands to tens of thousands of years, while groundmass alkali feldspar probably grew on timescales of decades to a few millennia.

While the literature has suggested that fast growth rates are needed to generate alkali feldspar megacrysts, we conclude that the formation of alkali feldspar megacrysts is controlled by the low number density of alkali feldspar crystals. This would be primarily obtained by low nucleation rates, which would be favored in a slowly cooling system, under low undercooling. We conclude that alkali feldspar megacrysts in the Cathedral Peak Granodiorite do not require special processes, and they are simply the consequence of late saturation of alkali feldspar in a large, slowly cooling silicic magma body.

## Data Availability

Data sets generated during the current study are available from the corresponding author on reasonable request.
